# Laboratory strains of *Bacillus anthracis* exhibit pervasive alteration in expression of proteins related to sporulation under laboratory conditions relative to genetically related wild strains

**DOI:** 10.1371/journal.pone.0209120

**Published:** 2018-12-17

**Authors:** Owen P. Leiser, Jason K. Blackburn, Ted L. Hadfield, Helen W. Kreuzer, David S. Wunschel, Cindy J. Bruckner-Lea

**Affiliations:** 1 Chemical and Biological Signature Science, Pacific Northwest National Laboratory, Richland, Washington, United States of America; 2 Emerging Pathogens Institute, University of Florida, Gainesville, Florida, United States of America; 3 Spatial Epidemiology & Ecology Research Laboratory, Department of Geography, University of Florida, Gainesville, Florida, United States of America; Loyola University Chicago, UNITED STATES

## Abstract

The spore forming pathogen *Bacillus anthracis* is the etiologic agent of anthrax in humans and animals. It cycles through infected hosts as vegetative cells and is eventually introduced into the environment where it generates an endospore resistant to many harsh conditions. The endospores are subsequently taken up by another host to begin the next cycle. Outbreaks of anthrax occur regularly worldwide in wildlife and livestock, and the potential for human infection exists whenever humans encounter infected animals. It is also possible to encounter intentional releases of anthrax spores, as was the case in October 2001. Consequently, it is important to be able to rapidly establish the provenance of infectious strains of *B*. *anthracis*. Here, we compare protein expression in seven low-passage wild isolates and four laboratory strains of *B*. *anthracis* grown under identical conditions using LC-MS/MS proteomic analysis. Of the 1,023 total identified proteins, 96 had significant abundance differences between wild and laboratory strains. Of those, 28 proteins directly related to sporulation were upregulated in wild isolates, with expression driven by Spo0A, CodY, and AbrB/ScoC. In addition, we observed evidence of changes in cell division and fatty acid biosynthesis between the two classes of strains, despite being grown under identical experimental conditions. These results suggest wild *B*. *anthracis* cells are more highly tuned to sporulate than their laboratory cousins, and this difference should be exploited as a method to differentiate between laboratory and low passage wild strains isolated during an anthrax outbreak. This knowledge should distinguish between intentional releases and exposure to strains in nature, providing a basis for the type of response by public health officials and investigators.

## Introduction

Bacteria growing in the laboratory experience dramatically different selective pressures than those found in the environment. *Bacillus anthracis* cells respond to conditions outside of mammalian hosts by forming a metabolically dormant endospore, capable of surviving extended periods of harsh conditions [1]. Cells must overcome interspecies competition and nutrient-limiting conditions to infect new hosts. In contrast to growth in the environment, growth conditions in the laboratory are often stable, with abundant nutrients–conditions tailored for optimum growth.

Intuitively, adaptation to different selective pressures between laboratory and environmental conditions will result in measurable genotypic or phenotypic changes. Indeed, long-term evolution has been studied extensively in an ongoing experiment in *Escherichia coli* [2–5], in which cultures have been maintained for over 60,000 generations with pervasive genomic and phenotypic changes observed. Additionally, Mikkola and Kurland [6], Eydallin et al. [7] and Saxer et al. [8] examined genomic signatures of adaptation of wild *E*. *coli* to laboratory conditions. However, far less is known about the mechanisms of wild pathogen adaptation to laboratory conditions: Sjödin et al. [9] investigated naturally occurring and laboratory strains of *Francisella tularensis* using whole-genome sequencing, and Leiser et al. [10] investigated the proteomic and genomic indicators of wild *Y*. *pestis* adaptation to laboratory conditions. Sjödin et al. examined very closely related strains of *F*. *tularensis* [9], and the laboratory-adapted strains of *Y*. *pestis* examined by Leiser et al. [10] were direct descendants of the respective starting wild strains. Systemic differences in gene/protein expression between wild and laboratory-adapted strains can be elucidated using genetically similar (same clade) but distinct (wild type or laboratory adapted) strains.

Previous work in our laboratory demonstrated the utility of proteomics to study mechanisms of *Y*. *pestis* adaptation to laboratory conditions [10, 11]. Here we broaden this work by investigating proteomic signatures of *B*. *anthracis* adapted to environmental and laboratory conditions. *B*. *anthracis* can survive for long periods of time in the environment in a metabolically dormant spore state, resulting in selection of wild isolates for growth almost exclusively in mammalian hosts. For this study, we selected seven temporally and geographically distinct wild isolates of *B*. *anthracis*, each genetically related to one of four laboratory strains (Ames, Sterne, Vollum, or Western North America), as well as the four laboratory strains themselves. All isolates were grown under identical conditions to compare protein expression differences resulting from long-term selective pressures between the two types. Our goal was to determine whether wild and laboratory strains being grown under identical conditions could be separated based on global protein expression.

## Results

### Global protein expression profiles differ between wild and laboratory strains

**Fig 1:**
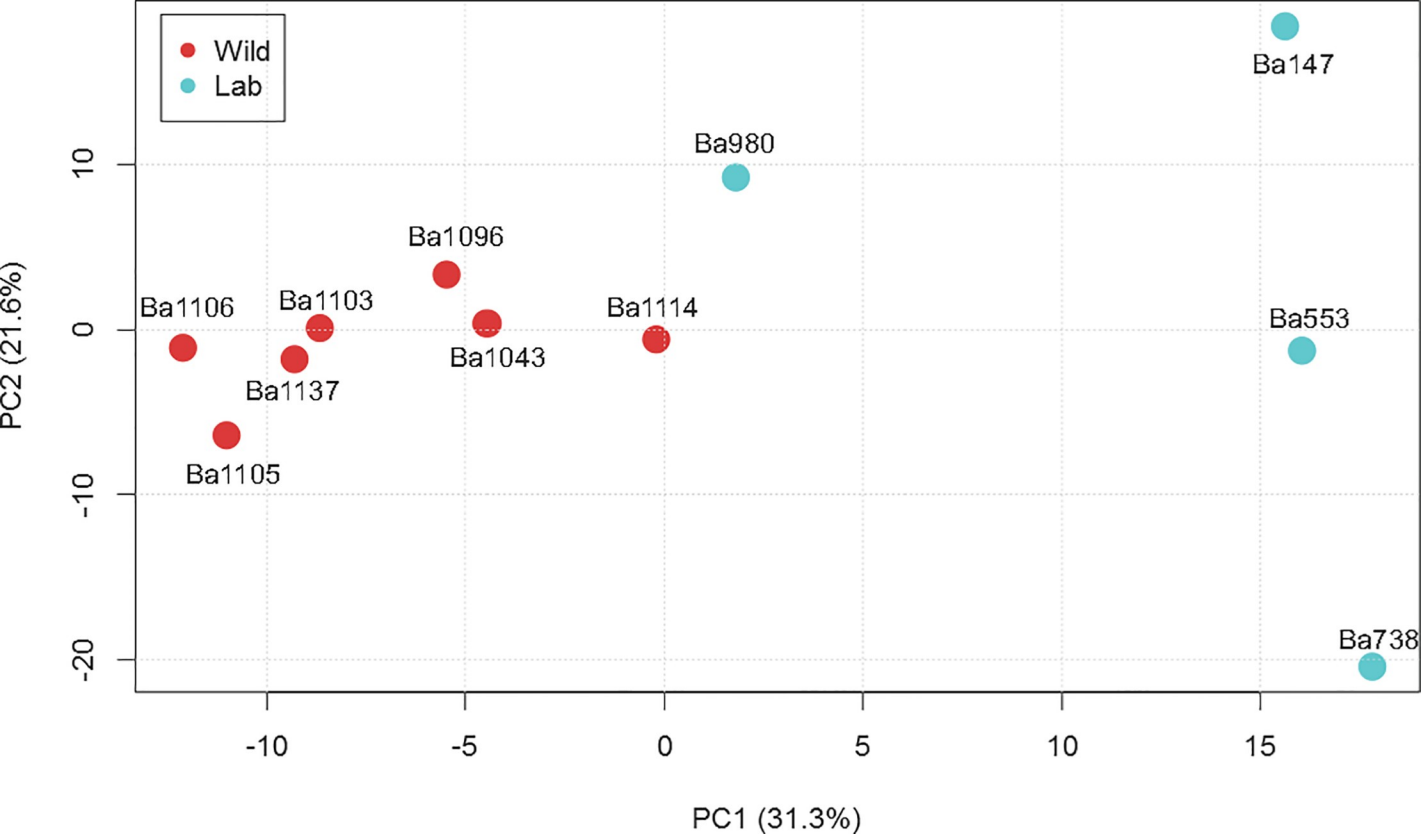
Principal coordinate analysis of protein abundance of all proteins identified in this study. Protein abundance values for wild (red) and laboratory (blue) strains of *B*. *anthracis* were averaged by strain prior to analysis.

In this study, we examined global protein expression differences between wild and laboratory strains of *B*. *anthracis* grown under identical experimental conditions. Using shotgun proteomics, we identified a total of 1,023 proteins across the 11 strains in the study. Of these, 96 proteins met our threshold for inclusion (ANOVA *q*<0.05, or present in only one class of strains) as differentially expressed between wild and laboratory strains. A full list of all observed proteins is available in S1 Table. Prior to any statistical treatment, we performed a simple principle component analysis (PCA) on all 1,023 observed proteins (Fig 1). Wild and laboratory strains were readily differentiated in this plot without any de-noising of the data. Interestingly, wild isolates cluster more closely together than their laboratory counterparts, especially Vollum (Ba980).

### Sporulation-related proteins make up large proportion of observed expression differences

Sporulation is the key strategy by which *B*. *anthracis* survives periods of nutrient scarcity. Wild isolates of this organism would be expected to experience higher pressure to sporulate in the natural environment, therefore, we surmised proteins related to sporulation would comprise a large proportion of those with differential expression between wild and laboratory strains. Indeed, of the 96 proteins with differential expression, nearly a third (*n* = 28; 29%) were shown to be directly or indirectly involved in the sporulation process (Table 1). We restricted assignment to proteins for which experimental evidence exists in the literature. With some exceptions, these proteins can be broadly assigned to three major categories: proteins whose expression is governed by the classical sporulation cascade, those regulated by AbrB and ScoC, and those whose expression is modulated by the CodY. Interplay between these regulons is complex, and some proteins are regulated by more than one circuit. Overall, these data suggest sporulation in wild *B*. *anthracis* cells is distinguishable from related laboratory strains.

**Table 1:** Identified proteins exerting direct or indirect effects on sporulation with significant expression differences between wild and laboratory strains.

Protein Name	Protein Description	Expression Ratio Wild:Lab
AbrB	Pleiotropic transition state regulator	2.02
AccB	Biotin carboxyl carrier protein of acetyl-CoA carboxylase	1.71
CitC	Isocitrate dehydrogenase	0.41
ClpP	Clp protease proteolytic subunit	2.39
CodY	Nutritional sensing pleiotropic regulator	0.65
CysK1	Cysteine synthase	1.73
EA1	S-layer protein	20.46
FabF	3-oxoacyl-ACP synthase	1.51
Fhs	Formate-tetrahydrofolate ligase	0.07
FtsZ	Cell division ring protein	1.46
GpsA	Glycerol 3-phosphate dehydrogenase	2.50
Hup-1	Signal recognition particle subunit	2.09
Hup-2	Histone-like protein	2.52
InfB	Translation initiation factor	1.67
Isp	Intracellular serine protease	3.72
IspG	1-hydroxy-2-methyl-2-butenyl-4-diphosphate synthase	0.55
MinD	Septum site determining protein	0.72
MurF	UDP-N-acetylmuramoyl-tripeptide D-analyl-D-alanine ligase	Wild only
OppC	Oligopeptide transport permease	0.34
PepF1	Oligoendopeptidase F	2.23
PhaR	synthase	2.51
RplD	Ribosomal protein L4	0.59
RplM	Ribosomal protein L13	1.52
RplS	Ribosomal protein L19	0.35
RplU	Ribosomal protein L21	0.48
ScoC	Global transcriptional regulator	1.72
Soj	Chromosome partitioning protein	1.59
Spo0A	Stage 0 sporulation response regulator	1.81
Spo0J	Stage 0 sporulation protein	Wild only
SpoIIE	Stage II sporulation serine phosphatase	Wild only
SpoVS	Sage V sporulation assembly protein	3.59
SsbB	Single-stranded DNA binding protein	1.83

### Canonical sporulation genes are more abundant in wild strains

Upon nutrient depletion, *Bacillus* spp. cells undergo a complex and ultimately irreversible process to form endospores capable of long-term survival in harsh environments [12, 13]. Sporulation is initiated by a phosphorylation cascade known as the phosphorelay, eventually resulting in an increase of phosphorylated Spo0A (Spo0A~P) which drives transcription from numerous downstream promoters [12]. Protein abundance data suggest this process in upregulated in wild strains. For example, Spo0A is 1.81-fold more abundant in wild strains, although its phosphorylation state was not determined in this study. Additional proteins directly involved in the sporulation process are also observed at higher levels in wild strains. Soj is a DNA-binding protein responsible for chromosome partitioning and also represses several Spo0A~P-dependent promoters [14] as well as *spo0A* transcription itself [15]. Soj activity is in turn repressed by Spo0J, likely by localization at the cell poles [14, 16]. Among the Spo0A~P-dependent promoters repressed by Soj is *spoIIE*, which is responsible for proper asymmetric septation during the early stages of sporulation [17]. SpoIIE is only observed in wild strains, suggesting that while wild Soj protein levels are 1.59-fold higher in wild strains, a concomitant increase in Spo0J (also identified in wild strains only) restrains its activity, resulting in at least partial derepression of Spo0A~P-dependent promoters. Indeed, several other proteins under positive regulation by Spo0A~P are observed at significantly higher levels in wild cells: PepF1 is present at 2.23-fold higher levels than laboratory strains, as are two proteins involved in fatty acid metabolism, PhaR and AccB (2.51- and 1.71-fold, respectively) and the dipeptide permease subunit DppE (3.45-fold). An additional protein, SpoVS, indirectly influences sporulation through motility-specific peptidoglycan hydrolases [18] and is present in wild strains at 3.59-fold higher levels.

### Protein abundance in metabolic circuits is consistent with upregulation of sporulation in wild strains

*Bacillus* spp., including *B*. *anthracis*, contain complex metabolic regulatory circuits governing response to nutrient starvation and virulence and interplay with the regulation of sporulation. CodY modulates one such circuit, and responds to intracellular concentrations of GTP and branched chain amino acids (BCAA) and influences pathogenesis and physiology [19–21]. The CodY regulon is well-characterized [22–25]. We observed an almost 40% reduction in CodY protein in wild strains relative to their laboratory cousins (expression ratio 0.65). Consistent with this observation, several proteins normally repressed by CodY are more abundant in wild strains. For example, DppE (dipeptide permease protein binding subunit) was present at 3.45-fold higher levels in wild strains. This is consistent with CodY suppression of the *dpp* operon [26]. Consistent with the findings of Chateau et al [23], we observed increased abundance of PepF1 (2.23-fold), CysK1 (1.73-fold), PhaR (2.51-fold), EA1 (20.46-fold), and AbrB (2.02-fold, discussed further below).

A second metabolic circuit with partial control over sporulation is modulated by AbrB and ScoC (also known as Hpr). AbrB is a transition-state regulator [27] and together with ScoC represses the oligopeptide transport operon *opp*, which is required for initiation of sporulation [28]. In our hands, both AbrB and ScoC are present at moderately higher abundance in wild strains (2.02- and 1.72-fold, respectively). In turn, we observed a single member of the *opp* operon, OppC at lower levels in wild than laboratory strains (0.34-fold).

### Accessory protein levels suggest increased sporulation in wild strains

In addition to those proteins directly controlling or falling within a regulatory circuit governing sporulation, many other proteins are known to be associated with sporulation through indirect metabolic effects, and their relative abundance provides circumstantial evidence of increased sporulation by wild strains of *B*. *anthracis*. For example, fatty acids are thought to provide both an energy source and a source of new membrane [29, 30] during sporulation. Together with increased levels of PhaR and AccB (under positive control of Spo0A~P), we observed increased levels of 3-oxoacyl-ACP synthase (FabF, 1.51-fold) and glycerol 3-phosphate dehydrogenase (GpsA, 2.50-fold), suggesting the importance of these molecules to wild cells.

We also observed differential expression of proteins required for appropriate chromosome segregation and cell division during sporulation. FtsZ is required for asymmetric cell division and localizes to the cell poles after Spo0A-dependent expression of SpoIIE [31], with its own expression driven by the sporulation-specific sigma factor σ^H^ [32, 33], and in wild strains is present at moderately higher (1.46-fold) levels than laboratory strains. MinD, a membrane-associated ATPase, inhibits polar cell division by binding Soj at the cell poles [34] and is less abundant in wild strains (0.72-fold). Soluble peptidoglycan precursors are an important prerequisite to σ^K^-dependent spore cortex formation [35] and are synthesized in part by MurF, which was identified only in wild strains. Finally, the DNA-binding protein Ssb-1 is present in 1.83-fold higher amounts in wild strains. In *Streptomyces coelicor*, this protein is implicated in chromosome segregation during sporulation [36], although similar studies have not been carried out in *Bacillus* spp.

Lastly, circumstantial support for the idea that wild cells more effectively sporulate is given by levels of certain ribosomal subunits. Ohashi et al. [37] used DNA microarray to examine genes associated with translation during sporulation. We identified four ribosomal subunits with differential expression between wild and laboratory strains: RplD, RplM, RplS, and RplU. Protein expression ratios (0.59, 1.52, 0.35, and 0.48, respectively) in this study were consistent with mRNA levels of the corresponding genes observed by Ohashi et al. in sporulating *B*. *subtilis* [37].

## Discussion

Adaptation of bacteria to laboratory conditions is an area of active research [5, 8, 10, 11]. Knowledge of the functional changes between wild pathogenic bacteria and laboratory adapted isolates have epidemiologic implications for categorization of an outbreak. In this study, we used proteomics to examine the differences in protein expression between common laboratory strains of *Bacillus anthracis* and related low passaged, wild isolates obtained from wildlife and livestock anthrax outbreaks in the western United States. Despite being grown in identical experimental conditions, laboratory strains of *B*. *anthracis* expressed a wide variety of proteins critical to or related to sporulation at lower levels than their wild cousins. In *B*. *anthracis*, transmission of spores in the environment is the key method to initiate disease in a host and is itself an area of active research [38–41].

**Fig 2:**
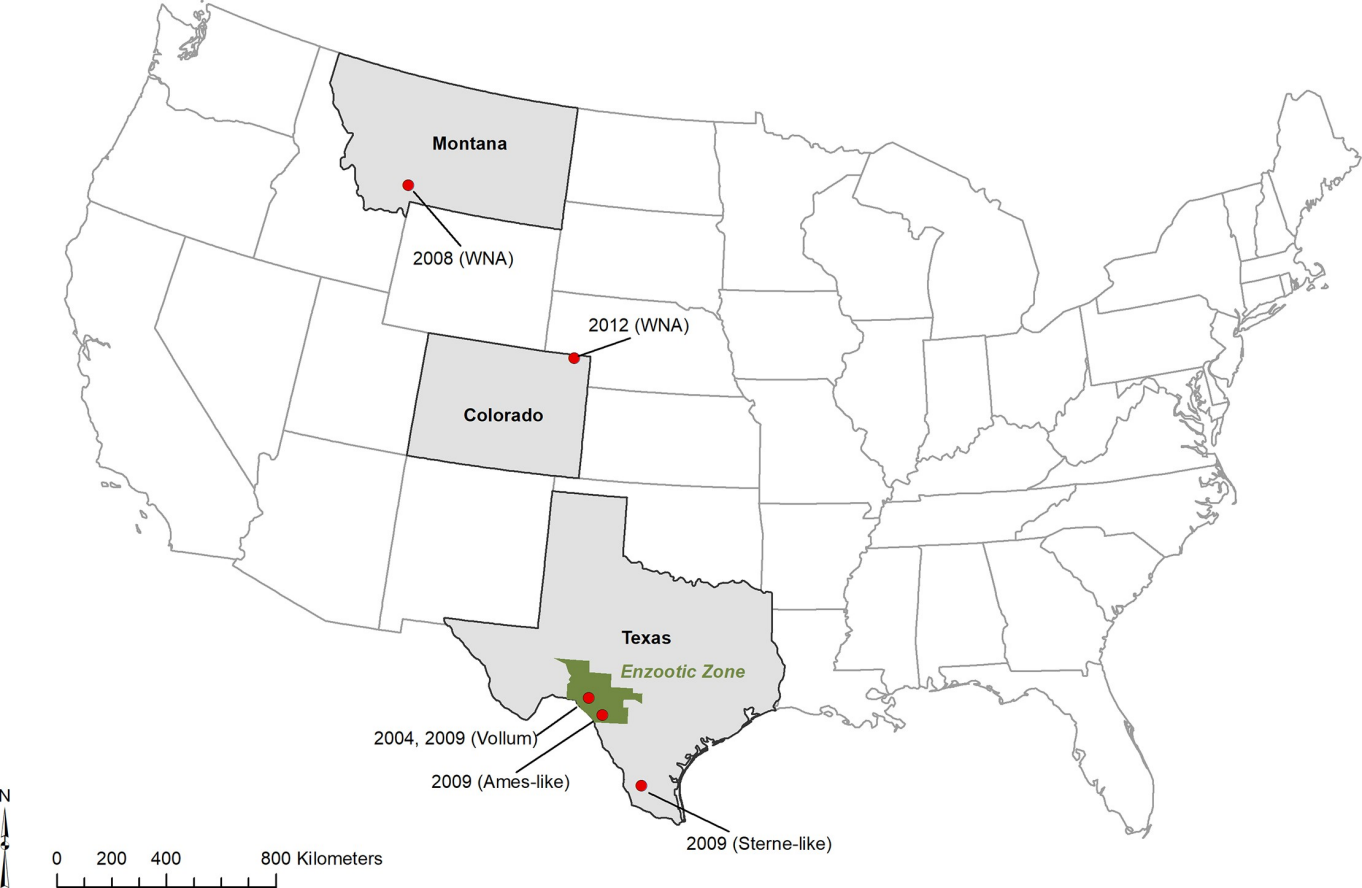
Geographic distribution of *B*. *anthracis* isolates used in this study. The green area denotes the Enzootic Zone of West Texas as defined by Blackburn et al. 2014 [42], where outbreaks are more frequent and typically larger than those of southern Texas.

The wild strains in this study were recovered across the geography of the active anthrax zone in the US [40], including the (re)emerging zone of Montana and Colorado, the enzootic zone of west Texas [42], and the sporadic zone of south Texas (Fig 2). Additionally, while all associated with animal deaths, these strains represent outbreaks of varying intensity. For example, the 2008 Montana strain was associated with an intense epizootic in multiple hosts and over 300 individuals [43], while the 2009 Texas Sterne-like strain was a small deer outbreak. These observations of protein pathways associated with sporulation were noted across host taxa and outbreak intensity, suggesting these pathways are required for “life in the wild” and reflective of *B*. *anthracis* and not any single lineage.

We observed evidence of pervasive alterations in the ability of laboratory strains of *B*. *anthracis* to sporulate, in the form of intricate interplay between the canonical “Spo,” AbrB/ScoC, and CodY regulatory circuits, as well as alterations in fundamental metabolism in support of sporulation. Sporulation is triggered by nutrient starvation, which initiates a phosphorylation cascade ultimately resulting in phosphorylation of the master regulator Spo0A~P [33]. While we did not specifically target the phosphorylated form of Spo0A, we observed evidence of its increased activity in wild isolates in the form of increased levels of Spo0A~P downstream targets: SpoIIE, PepF1, DppE, PhaR, and AccB (Table 1). Soj normally represses Spo0A~P activity by binding to target promoter regions, including *spoIIE* and *spo0A* [15], and is more abundant in wild strains. However, Soj is in turn repressed by Spo0J, which is observed only in wild strains, suggesting its activity is kept in check via a feedback inhibition mechanism. A related piece of evidence is the reduced level of MinD in wild strains. MinD is required for proper Soj localization and activity [34], and lesser amounts of MinD result in impaired repression of Spo0A~P.

In addition to the canonical regulation of sporulation, two other systems control expression of genes tied to the process: CodY and AbrB/ScoC (formerly Hpr). CodY is a master regulator of multiple responses in *Bacillus* spp., including the transition between exponential and stationary phase growth, nutrient sensing, competence, and virulence [21, 22]. It has also recently been shown to negatively regulate sporulation in *B*. *anthracis* [44]. We observed decreased levels of CodY in wild strains relative to laboratory, suggesting derepression of sporulation. Consistent with low levels of CodY in wild strains, we observed increased abundance of PepF1, CysK1, DppE, and EA1, which are all normally repressed by CodY. DppE expression is in turn positively regulated by Spo0A~P [45], indicating the multi-layered nature of sporulation regulation.

ScoC is a master regulator of sporulation initiation through catabolite repression [46] and controls expression of the App and Opp pentapeptide transport systems [47]. ScoC is negatively regulated by CodY [48]. In our analysis, ScoC levels were 1.72-fold higher in wild strains, with a concomitant decrease in OppC. It is not immediately clear why a decrease in proteins responsible for sporulation initiation was observed in wild strains; we speculate that these cells have already passed this checkpoint and therefore expression of initiation proteins was no longer required. The relationship between ScoC, AbrB, and Spo0A expression is similarly complex in these data. AbrB positively regulates ScoC expression [27] and is expressed at higher (2.02-fold) levels in wild strains than laboratory strains. AbrB in turn represses SpoIIE activity [49] and is repressed by Spo0A~P [50] and by autoregulation [51]. Such a complex regulatory framework has unknown influences on protein levels and suggests there is not one master regulator. However, the data are consistent with the hypothesis that sporulation as a process is upregulated in wild strains.

An additional line of evidence supports the hypothesis that wild strains of *B*. *anthracis* are more apt to sporulate than their laboratory cousins. We observed changes in basic cellular metabolic systems associated with sporulation. Specifically, we observed changes in fatty acid biosynthesis and cell division machinery. *De novo* fatty acid biosynthesis is required for efficient sporulation and is driven by Spo0A~P [29, 30]. Wild cells had increased levels of four key proteins in fatty acid biosynthesis directly controlled by Spo0A~P. AccB, the biotin carboxyl carrier protein of acetyl-CoA carboxylase, catalyzes the synthesis of malonyl-CoA as the first committed step of fatty acid biosynthesis [52]. FabF (3-oxoacyl-ACP synthase) and GpsA (glycerol 3-phosphate dehydrogenase) act downstream of AccB in discrete stages of fatty acid biosynthesis [29]. A fourth protein, PhaR (poly(3-hydroxybutyrate) [PHB] synthase) is more abundant in wild strains, indicating an increased need for carbon and energy storage molecules during the metabolically demanding process of sporulation [53]. An additional protein involved in fatty acid biosynthesis, AtoD was observed at increased levels in wild strains (S1 Table). However, its role in sporulation has not been established in the available literature.

Asymmetric cell division is a key step in sporulation by *Bacillus* spp. [13] and is inhibited in vegetative cells by the Min system (reviewed in [54]) including MinD. MinD normally localizes to the cell poles and attracts Soj [34] as a checkpoint to inhibit sporulation at inappropriate times in the cell cycle [13]. The Min system also positions FtsZ at the midpoint of the cell during vegetative cell division [54]. During sporulation FtsZ production is increased and it localizes to the cell poles in a σ^H^- and SpoIIE-dependent manner [31, 34]. Our findings are consistent with an enrichment for sporulating cells in wild strains: low levels of MinD would allow an increased amount of FtsZ to localize to the cell poles for effective prespore formation. Also consistent with increased spore formation in wild strains are 1) the observation of higher levels of SsbB (responsible for chromosome segregation during sporulation) [36], and 2) the identification of MurF (partially responsible for σ^K^-dependent accumulation of peptidoglycan precursors during spore cortex formation) [35] only observed in wild strains.

Finally, the relative abundance of four identified ribosomal subunits in wild strains is consistent with earlier work examining expression of the corresponding genes during sporulation [37]. Changes in global translation machinery suggest these cells have undergone significant metabolic alteration to sustain a sporulation phenotype even during growth in the laboratory.

Taken together, the data presented here suggest a global orientation of cellular metabolism towards sporulation in wild *B*. *anthracis* cells relative to genetically similar laboratory strains. If this response was the result of short-term adaptation to immediate growth conditions, we would not expect to see these protein expression differences when cells were grown under identical conditions. Rather, wild cells appear to be exquisitely primed to sporulate. The likely rationale for this adaptation is the fact that laboratory conditions are far removed from the selective pressures in the environment, and despite what we assume to be sound microbiological techniques on the part of researchers, laboratory strains inevitably responded to the selective pressures to their surroundings. In contrast, wild cells necessarily sporulate as a part of the host infection cycle and would be expected to efficiently sporulate since they have not had time to adapt to laboratory conditions.

This study provides a fundamental basis for discrimination between wild and laboratory strains of *Bacillus anthracis*. While there is no single “smoking gun” to indicate a sample is derived from wild vs. laboratory *B*. *anthracis*, comparison of protein levels in strains of known provenance could provide a first step towards classification. It will be necessary to empirically investigate the consequences of the observed differences in protein abundance between wild and laboratory strains of *B*. *anthracis* on sporulation, as hypothesis testing was beyond the scope of this study. Specifically, the hypothesis that wild strains more efficiently sporulate or sporulate sooner in BHI broth than laboratory strains remains to be tested. However, based on the pervasive alteration of metabolism in wild strains towards proteins related to sporulation, we speculate this will be the case.

## Materials and Methods

### Isolation of *B*. *anthracis* strains used in this study

*B*. *anthracis* strains used in this study were taken from the existing Martin E. Hugh-Jones *Bacillus anthracis* Collection housed at Emerging Pathogens Institute (EPI) at the University of Florida (Gainesville, FL USA; Martin E. Hugh-Jones *Bacillus anthracis* Collection). Geographically and temporally distinct wild strains of *B*. *anthracis* were isolated from wildlife during outbreak investigations in the western United States (Table 2 and Fig 2). Strains from Texas were collected from wild white-tailed deer (*Odocoileus virginianus*) that died of anthrax between 2004 and 2009. Strains from Montana were recovered during a large multi-species outbreak, including free ranging farmed bison (*Bison bison bison*) and wild elk (*Cervus canidensis*) [43]. An additional strain was isolated from a domestic cow in northwestern Colorado; the first report of anthrax in that area since the late 1970s. Wild strains were genotyped using multi-locus variable number tandem repeat (MLVA) analysis following Blackburn et al. [55] and paired with genetically similar laboratory strains for comparison of protein abundance. Wild isolates were cultured on 5% sheep blood tryptic soy agar (SBA) prior to this study.

**Table 2:** *B*. *anthracis* strains used in this study.

Strain ID	Wild/Laboratory	Related Laboratory Strain	Strain Details
Ba553	Laboratory–Sterne	NA	NA
Ba738	Laboratory–Ames	NA	NA
Ba980	Laboratory–Vollum	NA	NA
Ba147	Laboratory–WNA	NA	NA
Ba1114	Wild	Sterne^1^	2009 Texas deer
Ba1105	Wild	Ames^2^	2009 W. Texas deer
Ba1106	Wild	Ames^2^	2009 W. Texas deer
Ba1096	Wild	Vollum (A4)	2004 W. Texas deer
Ba1103	Wild	Vollum (A4)	2009 W. Texas deer
Ba1137	Wild	WNA (A1.a)	2012 Colorado cow
Ba1043	Wild	WNA (A1.a)	2008 Montana elk

^1^MLVA-based genotype relates to Sterne based on lack of pX02 plasmid

^2^Ames-like lineage but not true Ames

### Growth and inactivation of cells

All culturing and handling of live *B*. *anthracis* cultures was performed in the Biosafety Level 3 (BSL3) facility at EPI. Use of live *B*. *anthracis* strains for this study was approved by the University of Florida institutional biosafety committee and environmental health and safety. Samples were passaged once on Tryptic Soy Agar prior to inoculation into 20 mL of brain-heart infusion broth (BHI, BD Difco). Cultures were grown overnight with vigorous aeration prior to harvesting; this method yielded approximately 10^7^ CFU/mL per culture. Cells were pelleted by centrifugation, resuspended in 1–2 mL fresh BHI, and autoclaved on a liquid cycle with 90-minute exposure to wet steam. Samples were tested for sterility prior to shipment to PNNL for proteomic preparation and analysis.

### Proteomic sample preparation and LC-MS/MS

Unless otherwise noted, all chemicals used during sample preparation were of analytical grade, and all chemicals used during liquid chromatography tandem mass spectrometry were of mass spectrometry grade. Raw proteomic data were deposited into the PRIDE database [56, 57] under accession number PDX010120.

Samples were separated into randomized blocks for preparation, reflecting the overall proportion of wild and laboratory strains, to minimize confounding effects during statistical analysis. Sample preparation was carried out essentially using the method of Deatherage Kaiser et al [58]. 100 μL of each sample was pelleted by centrifugation at maximum speed for 5 minutes and resuspended in 500 μL freshly prepared ice-cold 20% w/v trichloroacetic acid (TCA, Sigma). Resuspended cells were incubated at -20°C for 24 hours. Samples were pelleted by centrifugation at maximum speed for 5 minutes at 4°C, and washed twice with 200 μL ice-cold acetone. Excess acetone was drawn off and samples were dried in a Vacufuge Plus (Eppendorf) under vacuum without heat for ~5 minutes.

Dried samples were resuspended by pipetting and vortexing in 100 μL lysis buffer (8 M urea [Sigma]; 50 mM triethylammonium bicarbonate [Sigma], pH 8.5; 14.3 mM β-mercaptoethanol [Sigma]) and gently centrifuged to remove liquid from the sides of tubes. Resuspended samples were incubated at 60°C for 1 hour with shaking in a ThermoMixer (Eppendorf). Iodoacetamide (Thermo) was added to a final concentration of 1.5 mM to alkylate cysteine residues, and samples were mixed by vortexing and gently centrifuged. Samples were incubated in the dark at 37°C for 30 minutes with shaking. After alkylation samples were diluted 10-fold in 50 mM ammonium bicarbonate (Sigma) and calcium chloride (VWR) was added at a final concentration of 1 mM. Samples were mixed thoroughly by vortexing and centrifuged gently to remove liquid from the sides of tubes. 4 μg Trypsin Gold (Promega) was added to each sample and mixed by pipetting. Samples were digested overnight at 37°C with gentle (~200 rpm) shaking.

After digestion samples were acidified with trifluoroacetic acid (TFA, Sigma) at a final concentration of 0.1% v/v. Solid phase extraction (SPE) was performed with a vacuum manifold using Strata C-18T columns according to the manufacturer’s instructions. Briefly, 1 mL of 100% methanol (Sigma) was added to activate the resin, followed by a conditioning rinse using 1 mL 0.1% v/v TFA in water. Samples were added and passed through the resin, followed by a rinse with 1 mL 0.1% v/v TFA/5% v/v acetonitrile (ACN, Sigma) in water. Peptides were eluted from SPE columns into clean 1.5 mL low-protein binding tubes (Fisher Scientific) using 1 mL 0.1% v/v TFA/80% v/v ACN in water. ACN was removed and samples brought to ~100 μL using a Vacufuge Plus (Eppendorf).

Peptide concentration was determined using BCA assay (Pierce). Peptides were diluted to ~1 μg/μL in 0.1% v/v formic acid (EMD) and transferred to high performance liquid chromatography (HPLC) vials with inert glass inserts, sealed with screw caps, and stored at -20°C until analysis. Each biological replicate was injected in duplicate, using a randomized run order for each block. Peptides were separated using HPLC on an Agilent Infinity 1260 HPLC system. The column was a fused silica capillary (40 cm x 150 μm inner diameter) packed with 5 μm particle size, 300 Å pore size Jupiter C18 resin (Phenomenex). 1 μg peptides were injected and subjected to the following 160-minute gradient: 100% Solvent A for 10 minutes; 0%-7.5% Solvent B over 1 minute; 7.5%-45% Solvent B over 109 minutes; 45%-95% Solvent B over 2 minutes; 95% Solvent B for 10 minutes; 95%-0% Solvent B over 4 minutes; 100% Solvent A for 23 minutes. Solvent A was 5% v/v ACN/0.1% v/v formic acid and Solvent B was 95% v/v ACN/0.1% v/v formic acid. Blanks consisting of 5 μl injections of 50% v/v isopropanol/50% v/v acetone/0.1% v/v formic acid were run with a shorter gradient between samples to minimize column carryover.

The HPLC was coupled to a Thermo Scientific LTQ Orbitrap XL mass spectrometer via a custom electrospray emitter consisting of an etched fused silica capillary [59]. The mass spectrometer was operated in data dependent “high-low” mode with a high-resolution (*R* = 30,000) precursor scan collected in the Orbitrap followed by collision-induced dissociation (CID) fragment scans of the top seven most intense precursors collected in the ion trap. Data dependent acquisition parameters were: dynamic exclusion repeat count 2, repeat duration 30 seconds, exclusion list size 250, exclusion list duration 180 seconds.

### Proteomic data analysis

Relative protein abundance was determined using label-free quantification (LFQ) functionality of MaxQuant v1.5.3.30 [60, 61], using match between runs and requantify options. All other MaxQuant settings were left at default values. Translated *B*. *anthracis* genomes for Ames Ancestor (accession 261594.21), Sterne (accession 260799.41), Vollum (accession 261591.12), and Western North America (WNA, accession 212045.4) were downloaded from PATRIC [62] on 2/3/16. All LC-MS/MS output spectra were searched against each database separately. After MaxQuant LFQ a custom R script was used to ensure differences in protein names between genomes, as well as subtle differences in protein sequence between genomes, did not artificially influence protein abundance measurements across genomes. Briefly, this script first retrieved protein abundance values from a given set of samples (e.g. Ames-related strains from the Ames Ancestor database search, Sterne-related strains from the Sterne database search, etc.). BLASTp [63] was then used to match protein sequences annotated in the Sterne, Vollum, and WNA genomes to those in Ames Ancestor. Finally, protein abundance values for Sterne, Vollum, and WNA proteins were matched to corresponding Ames Ancestor proteins and output to a single file.

To investigate protein abundance differences between wild and laboratory strains of *B*. *anthracis*, as opposed to abundance differences between individual cultures, all biological and technical replicates for each strain were averaged. Statistical analysis of average protein abundance between wild and laboratory strains was carried out using Inferno (http://omics.pnl.gov/software/infernordn), a freely available version of DAnTE [64]. Proteins were judged to have significantly changing abundance if the *q*-value from ANOVA (comparing all wild to all laboratory strains) was less than 0.05, or if the protein was detected in only one category, and in more than half of the strains for that category. Proteins were assigned to functional categories using KEGG [65] and eggNOG [66].

## Supporting information

S1 Table Raw protein abundance data in tabular format. (XLSX)
